# Pharmaceutical and Pharmacological Aspects of Modulation of Oxidative Stress 2020

**DOI:** 10.1155/2022/9842198

**Published:** 2022-01-27

**Authors:** Luciano Saso, Sasanka Chakrabarti, Elzbieta Miller

**Affiliations:** ^1^Department of Physiology and Pharmacology “Vittorio Erspamer”, Sapienza University of Rome, 00161 Rome, Italy; ^2^Department of Biochemistry, Maharishi Markandeshwar Institute of Medical Sciences and Research, Mullana, Haryana, India; ^3^Department of Neurological Rehabilitation, Medical University of Lodz, Poland

Oxidative stress, though not really on the driver's seat, continues to play key roles within the pathogenic spectrum of many diseases especially cancer, inflammatory, and degenerative diseases. Thus, reactive oxygen species- (ROS-) mediated tissue injury and redox signaling are intensely investigated in disease conditions and model systems for a better understanding of the pathogenesis and identification of new drug targets.

The special issue contains 29 articles which describe the involvement of oxidative stress in various clinical conditions and experimental models of diseases, drug actions, and environmental injuries. We can summarize some of the important concepts and results that have emerged from these studies. In the pathogenesis of AD, a body of evidence links the process of neuroinflammation with the increased production of ROS by microglial NADPH oxidase, and this suggests a potential role of NADPH oxidase inhibitors as disease-modifying agents for Alzheimer's disease. U. Ganguly et al., in their review succinctly evaluated the current state of research on NADPH oxidase inhibitors [1]. The important role of oxidative stress and mitochondrial dysfunction in ALS and positive effects of antioxidants in preclinical trials of this disease have been reviewed by T. Cunha-Oliveira et al.; the authors suggested that oxidative stress-related damage mechanisms could be different in different subtypes of ALS having defined mutations, and thus, specific and targeted antioxidant therapy would be needed for different subtypes of ALS for novel and effective therapeutic interventions [2]. In an exhaustive review on the complex mechanisms of atherogenesis, C. Khatana and coauthors highlighted the special role of oxidized-LDL and the crucial involvement of antioxidant enzymes in this process [3]. The authors also discussed the various therapeutic options including the use of antioxidants and life-style management to combat the problem of atherosclerosis and its complications. A rather unusual but elaborate review on the molecular hydrogen as a multipotential therapeutic agent for a variety of diseases including COVID-19 has been presented by M. Yang et al., with a load of direct and indirect experimental evidence of hydrogen acting as an antioxidative or anti-inflammatory agent or a modulator of mitochondrial functions, cell death pathways, and many other biological processes [4].

The research articles contained in this special issue examined a wide range of interesting questions related to redox pathophysiology, and we may mention a few of them here just to indicate the diverse nature of these studies. A. Abbasi and others showed how oxidative stress could be detrimental to cancer cells by causing a loss of ionic homeostasis through alterations in the activities of pumps and channels; hyaluronic acid apparently can be used to optimize and target the cytotoxic effects of oxidative stress on cancer cells [5]. The poor prognosis of acute myeloid leukemia (AML) has been attributed to enhanced ROS production; D. Zhang et al., showed using cell-based and animal studies that azelaic acid could impact against AML by upregulating the peroxiredoxins (Prdxs2/Prdx3) with consequent scavenging of ROS [6]. Likewise, the protection of LPS-induced acute kidney injury and ischemia/reperfusion mediated cardiac damage by dexmedetomidine and Klotho protein, respectively, indicated the therapeutic potentials of these molecules which were partly attributable to their antioxidative functions [7,8]. An interesting clinical study by S. Ahmed et al. indicated the potentiation of antiviral therapy in patients of hepatitis C by cotreatment with black cumin and ascorbate which decreased the oxidative stress and viral load and improved several clinical parameters [9]. Nrf-2 activators have been under scanner for quite some time as potential drugs for a variety of disease conditions where oxidative stress and inflammation are involved. A significant research paper in this issue attempted to explore the reasons for unexpected toxic side effects of a potent Nrf-2 activator called bardoxolone methyl, and the authors showed that the drug caused severe mitochondrial bioenergetic impairment along with apoptosis and necrosis of endothelial cells in a dose-dependent manner which could be related to its toxicity [10]. There are many other interesting articles in this issue which we would not discuss individually, but together, they send out two important messages. Firstly, oxidative stress affects specific aspects of different diseases or environmental toxicities which could be identified by studies in different experimental models. Secondly, attempts should be continued to develop new drugs targeting oxidative stress which should be safe and of higher efficacy or bioavailability, but in most cases, such drugs, though clinically relevant, would be a part of an add-on or adjunct therapy for a disease condition.

*Luciano Saso* *Sasanka Chakrabarti* *Elzbieta Miller*

